# A proposal to embed patient and public involvement within qualitative data collection and analysis phases of a primary care based implementation study

**DOI:** 10.1186/s40900-023-00440-7

**Published:** 2023-05-31

**Authors:** Alice Moult, Carmel McGrath, Kate Lippiett, Caroline Coope, Simon Chilcott, Cindy Mann, Nicola Evans, Andrew Turner, Krysia Dziedzic, M. C. Portillo, Rachel Johnson

**Affiliations:** 1 Impact Accelerator Unit, Keele University, Newcastle-Under-Lyme, ST5 5BG UK; 2 NIHR Health Protection Research Unit in Behavioural Science and Evaluation, Population Health Sciences, Bristol Medical School, University of Bristol, Bristol, UK; 3 The National Institute for Health and Care Research Applied Research Collaboration West (NIHR ARC West), University Hospitals Bristol and Weston NHS Foundation Trust, Bristol, UK; 4 Faculty of Health and Applied Sciences, School of Health and Social Wellbeing, University of West England, Bristol, UK; 5 School of Health Sciences, NIHR ARC Wessex, University of Southampton, Southampton, UK; 6 Centre for Academic Primary Care, Bristol Medical School, 39 Whatley Road, Bristol, UK; 7 National Institute for Health Research Applied Research Collaboration West (NIHR ARC West), University Hospitals Bristol and Weston NHS Foundation Trust, Bristol, BS1 2NT UK; 8 Centre for Academic Primary Care, University of Bristol, Bristol, BS8 2PS UK

**Keywords:** Patient and public involvement, Qualitative, Data collection, Data analysis

## Abstract

**Background:**

Patient and public involvement (PPI) is increasingly seen as essential to health service research. There are strong moral and ethical arguments for good quality PPI. Despite the development of guidance aimed at addressing the inconsistent reporting of PPI activities within research, little progress has been made in documenting the steps taken to undertake PPI and how it influences the direction of a study. Without this information, there are minimal opportunities to share learnings across projects and strengthen future PPI practices. The aim of this paper is to present details on the processes and activities planned to integrate PPI into the qualitative research component of a mixed-methods, multi-site study evaluating the implementation of a smart template to promote personalised primary care for patients with multiple long-term conditions.

**Methods:**

This proposal describes the processes and activities planned to integrate PPI into the development and piloting of qualitative data collection tools (topic guides for both practice staff and patients) and a tailored data analysis package developed for PPI members incorporating broad concepts and specific methods of qualitative data analysis.

**Discussion:**

Outputs relating to PPI activity may include clear, concise and suitably worded topic guides for qualitative interviews. Piloting of the topic guides via mock interviews will further develop researchers’ skills including sensitisation to the experiences of participants being interviewed. Working with PPI members when analysing the qualitative data aims to provide reciprocal learning opportunities and may contribute to improving the overall rigour of the data analysis. The intent of publishing proposed PPI activities within this project is to inform the future delivery of high quality PPI.

**Supplementary Information:**

The online version contains supplementary material available at 10.1186/s40900-023-00440-7.

## Abstract (plain language summary)

Patient and public involvement (PPI) improves healthcare research, however, there is little published evidence of proposed PPI activities within a research study. The aim of this article is to describe the proposed PPI activities which are to be integrated into a study implementing a smart template to promote personalised care for people with multiple long-term conditions within primary care in the United Kingdom. The proposal describes the ways in which PPI members will be included within the data collection and analysis phases of a research study which includes interviews with primary care staff and patients. PPI members will be asked to develop questions for these interviews and to take part in a mock interview whereby a researcher interviews a PPI member. The proposal also provides details on qualitative data analysis workshops which will be specifically developed for PPI members. The impact of PPI activities could include clear, concise and suitably worded questions used within the interviews. Piloting of these questions via mock interviews may enable researchers to further develop their interviewing skills. It is anticipated that involving PPI members when analysing qualitative data will provide opportunities for reciprocal learning and lead to rich interpretations of the data, inclusive of the PPI members’ perspectives. Publishing a record of planned PPI activities and potential impacts demonstrates the rationale and considerations made by the team to ensure that involvement in this study is meaningful and has potential benefits for all involved. The team hopes this proposal will support others with the planning and delivery of PPI activities. In future publications, we will reflect on the learnings, challenges, and outcomes from the PPI activities detailed in this proposal.

**Supplementary Information:**

The online version contains supplementary material available at 10.1186/s40900-023-00440-7.

## Background

Patient and Public Involvement (PPI) in research can be defined as research carried out “with” or “by” patients and public contributors rather than “to”, “about” or “for” them [1]. PPI is put into practice through people with lived experience of healthcare discussing, helping to make decisions and conducting research in order to enhance study relevance, design, recruitment, data analysis, reporting and governance [2–4]. There are strong moral, ethical and political arguments for involving people with experiential knowledge in research [5, 6]. The moral and ethical rationales are based on the democratic principles of transparency and accountability; those affected and paying for the research have a right to be involved and have a voice in how and what research is conducted. The political reasons relate to the policy and funding requirements to involve patients and the public in research. Within the United Kingdom (UK), the National Institute for Health and Care Research (NIHR) is the major funding body for health research and requires researchers when applying for funding to describe how they have involved the public in the design and planning of the project as well as plans for further involvement throughout the project [7].

Involving PPI members can enhance qualitative research methods [8]. Qualitative data collection can be aided by PPI members contributing to topic guides and providing feedback on interviewing techniques [9, 10]. PPI members can add to the credibility of qualitative data analysis by ensuring pertinent themes which represent public members’ perspective are identified [11, 12]. When integrating PPI into any phase of a study it is important to consider the skills required of PPI members for meaningful involvement. Providing or offering a training and data analysis workshops may help PPI members to feel supported to access and engage in PPI activities [13]. Within the context of mental health research, Lovell and colleagues aimed to enhance PPI members’ knowledge by providing a tailored training course on research methods [14]. Although all PPI members reported an increased understanding of both qualitative and quantitative methods, the course structure and content were not described. Whilst the importance of involving PPI members within qualitative research methods is evident, there is little description as to how PPI members are included. Access to a knowledge base reporting how research teams have included PPI within qualitative data collection and analysis may inform future delivery of such activities and identify best practice within this area.

Despite developments in both addressing the inconsistent reporting of PPI activities and producing guidance to help researchers plan and conduct meaningful PPI [1, 11, 15–17], little progress has been made in the documentation of proposed PPI activity. Whilst PPI is not research, a proposal reporting planned PPI activities may be of great significance to the researchers conducting the study and the wider academic community. The proposal could determine the specific areas of PPI activities, state the aims and methods of involvement and describe the proposed PPI impact. The process of writing a proposal allows researchers to plan and review planned PPI activities and also provides a basis in which to evaluate and reflect upon PPI activities. The wider academic community and PPI members could also benefit from the publication of PPI proposals as they provide guidance and inspiration for conducting future PPI activities.

The objective of this article is to describe proposed PPI activities relating to qualitative data collection and analysis which is embedded throughout an on-going research project titled ‘Personalised Primary care for Patients with Multiple long-term conditions (PP4M)’.

## Methods

### The role of PPI within the personalised primary care for patients with multiple long-term conditions study

The PP4M study aims to support and evaluate the implementation of a smart template for use by primary care staff to promote personalised care for patients with multiple long-term conditions. The PP4M study will investigate barriers and facilitators of implementation, and intends to provide evidence of impact in meeting the aim of providing more personalised care. The PP4M study is being conducted across three regions in the UK; Bristol, Keele and Southampton. Researchers within the PP4M study will work with PPI members in each region at every stage of the study. The study is a collaboration between four NIHR Applied Research Collaborations (ARCs): ARC West, West Midlands, Wessex, and South West Peninsula. Each region has their own PPI group supported by the regional NIHR Applied Research Collaborations (ARCs). The role of an ARC is to support health and care research.

The PP4M study incorporates a range of qualitative and quantitative data collection methods, however, the scope of this proposal focuses on PPI activity within the qualitative component of the study. Qualitative data will be collected from participating general practices from the three regions. The study plans to conduct interviews with patients and staff members, and to video-record patient consultations.

- *Patient interviews:* Patients will be invited to participate in interviews about their experience of care in general practice for their long-term health conditions, and about their experience of their consultations after the template has been introduced.
- *Staff interviews:* Clinical staff involved in using the template, and non-clinical staff involved in organising the reviews, will be invited to take part in an interview.
- *Recorded consultations:* A sample of patient and clinician pairs will be invited to participate in a recorded observation of their review consultation at which the new template is used, to understand the part it plays in the review and the interaction between the clinician and patient. The patients and staff taking part in recorded observations will also be invited to be interviewed and form part of the sample described above.

### Proposed PPI objectives

Researchers aim to integrate PPI perspectives into the qualitative data collection and analysis. The PPI objectives within this study are:

- To co-produce and pilot separate topic guides for clinical staff, non-clinical staff and patients to ensure that they are clear, concise and suitably worded.
- To identify and address PPI members’ training needs with regards to qualitative data analysis which will provide them with the necessary skills to contribute meaningfully to the analysis.
- To collaborate with PPI members when interpreting and refining the qualitative data analysis, particularly focusing on the barriers and facilitators of implementing a smart template to promote personalised care for patients with multiple long-term conditions.

### Approach to PPI activities

A PP4M PPI steering group has been convened prior to the start of this study. The steering group compromises of academics and PPI members from all three regions. Each region also has a local PPI group which meet to discuss the study. At the beginning of the PP4M study the PP4M PPI steering group met to decide to what extent the PPI involvement would be. The steering group decided that a co-productive approach to PPI activities will be taken. Co-production is a specific approach to PPI whereby researchers and the public share power and decision making [18]. A key principle of co-production is reciprocity, which is at the centre of this PPI proposal. Public contributors will be gaining new knowledge, if they wish to, along with helping to develop data collection tools and shape the analysis [19]. Whilst some research has suggested a cautious approach to co-production [20], academic researchers and PPI members thought this approach was important to provide a voice to individuals experiencing multiple long-term conditions. The steering group co-produced an over-arching plan for how PPI will be integrated throughout the study (see Additional file 1). A Research Fellow in Public Involvement (CMcG) will play a key role in guiding the academic researchers to meet the proposed objectives. CMcG will also work with the PPI members to ensure that they are supported throughout the proposed PPI activities by referring to best practice guidance including the UK Standards for Public Involvement [21]. PPI members will be offered reimbursement for their time and any travel expenses.

### Recruitment of PPI members

Due to the PP4M study focusing on the management of multiple long-term conditions within primary care, PPI members with lived experiences of multiple long-term conditions, or with recent experience of primary care services, were sought; these individuals will have lived experiences which are specific to the PP4M project.

The recruitment of public contributors into the local PPI groups differed in each region. Members of the Keele PPI group were recruited through a Research User Group (RUG) hosted by the University’s Impact Accelerator Unit. To ensure diversity within the RUG a Race Equality Ambassador works with seldom heard groups with the vision to invite them to be part of the RUG. In Southampton, public contributors were recruited through the ARC PPI group. In Bristol an advertisement was sent out through local public involvement mailing lists including the People in the Health West of England [22]. This network is a regional collaboration led by the University of the West of England. It is a network that brings together key research partners and public contributors from across the NIHR and beyond to work jointly on public involvement. CM reviewed the responses, seeking to recruit individuals with diverse backgrounds and lower socio-economic status who had provided a brief self-description and reasons for wanting to be involved.

CMcG will capture demographic information of all PPI members who contribute to any activities relating to qualitative data collection and analysis. PPI members will be asked to provide self-selected characteristics and this information will be anonymous and kept confidential. Only academic members of the research team will be able to access this information. The researchers will use this data to reflect on whether PPI activities were inclusive to seldom-heard groups; such groups are not often involved in PPI activities.

### Prior PPI activities

PPI members are considered key members of the research team and have already provided significant input into project design and evaluation measures. Alongside the specific activities described below PPI members also provide ad hoc input on issues that arise during the course of the study.

### Developing and piloting the topic guides

The academic members of the research team have developed separate topic guides for clinical staff, non-clinical staff and patients (three topic guides in total). Each of the three local PPI groups will focus on one topic guide within their PPI activities. Academic researchers will meet to decide which different topic guide each local PPI group will focus on. Academic members of the research team will then meet with PPI members within each region to discuss the chosen topic guide. Within this meeting the group will nominate one member who be involved in a pilot interview. To resemble the interview data collection methods in the study, only one public contributor will be invited to a pilot interview.

The PPI members from each region will be sent their allocated topic guide via email or post. Guidance notes will be sent along with the topic guide with the aim of providing instructions to the PPI members. The guidance notes will provide a brief explanation of PP4M and outline the nature and aims of the PPI activity (e.g. to ensure topic guides are clear, concise and suitably worded). Within the notes PPI members will be informed that they will be asked to read each question and to provide their feedback.

An academic researcher from the study team will then meet with the PPI members for approximately one hour to discuss and make amendments to their allocated topic guide. PPI members will be given the choice as to whether they would prefer an in-person or virtual meeting. Following the meeting PPI members will be encouraged to provide additional feedback by contacting the researcher via email or telephone. Ensuring that the topic guide still meets the study’s research aims, the researchers will make any reasonable amendments to the topic guides based on feedback received from PPI members. The researchers will report back to the PPI members on how they have shaped the topic guides via email or telephone.

Once the amendments to the topic guides have been made, a researcher from each region will conduct one pilot interview either in-person or virtually, using their chosen topic guide, with one PPI member. Each local PPI group will nominate one member to be interviewed. The nature of the pilot interview will be dependent on which topic guide is being piloted. The PPI member will assume the role of patient when piloting the patient topic guide. Clinical and non-clinical academic researchers will be interviewed by the public contributor when piloting the clinical and non-clinical staff’s topic guide. The pilot interview will last approximately one hour. After the pilot interview, there will be time for the PPI member to provide advice on the questions within the topic guide and on how the interview was conducted. Again, PPI members will be encouraged to provide feedback outside of the meeting and the researchers will report back to the PPI members on how their comments have influenced the topic guides.

After the development and piloting of the topic guide has been completed, each local PPI group will meet to reflect, discuss and to provide feedback on how they thought the PPI activity went. Academic researchers from each region will make notes of any feedback received.

The academic researchers decided that they would not ask PPI members to conduct interviews within the data collection phase of the project. The researchers thought this would be burdensome to the public contributors.

### Qualitative data analysis

To achieve the PPI objectives, three cross-region qualitative data analysis workshops will be held. The PP4M PPI steering group (including researchers and PPI members) will develop the content of the workshops. The workshops will be created based on the needs of PPI members. Prior to the workshops CMcG will create a short survey to identify skills and knowledge needs of each PPI member. Within the survey CMcG will provide some learning anchors such as ‘An introduction to Qualitative methods’ or ‘The difference between Qualitative and Quantitative methods’, but the PPI members will be encouraged to express other training needs if these anchors are not representative of their views.

### Content of the workshops

- Workshop one: “An introduction to qualitative data analysis”
  An advertisement will be distributed to all PPI members inviting them to a training workshop based on qualitative research methods. The training will be broad in nature and include practise analysis of a sample transcript derived from two mock interviews conducted by SC, the PPI co-applicant, interviewing CM, based on the topic guides for patients and for health professionals. The workshop will be provided by CMcG, AM, KL and CC.
- Workshop two: “Helping the researcher to interpret the data: Part 1”
  PPI members from each region will be invited to participate in a workshop to provide their interpretations of the developing analysis. Researchers from each of the regions will virtually present their developing analysis and anonymised quotations. PPI members will be invited to share their thoughts and interpretations of the data.
- Workshop three: “Helping the researcher to interpret the data: Part 2”
  Once the researchers have reached a more advanced stage of the analysis, PPI members will be invited again to comment upon the analysis. This will involve researchers presenting the analysis to PPI members, receiving feedback and making any changes.

The three workshops will be recorded, verbal consent from PPI members will be obtained. After each workshop CMcG and AM will watch the recordings and write detailed notes. The recordings of the workshops will then be deleted. The notes from each workshop will be sent to the PPI members who attended that workshop and researchers conducting the qualitative analysis. Each of the workshop sessions will be evaluated by PPI members using an online version of the Cube Evaluation Framework [23]. The framework evaluates PPI across four dimensions: voice (the extent to which contributors feel they have a weak or strong voice in decision-making); contribute (the number of ways to get involved to accommodate different contributors' needs); agenda (the balance between organisation and public contributor concerns); change (the willingness or resistance to change by the organisation) [24]. Researchers will complete an impact log following each workshop [25]. The impact log will capture the date of activities, who attended, a brief description of the PPI activities, impact of PPI and any other comments the academic researchers deem appropriate. Supplementary materials, such as presentations developed by the researchers, and the offer of a pre-meeting with a researcher, will be provided before each workshop to provide any additional support if required.

### Feedback for PPI members

Within Workshop two, PPI members will help researchers to interpret and analyse the qualitative data. After Workshop two CMcG, AM, CC and KL will reflect on their own and PPI members’ interpretations of the quotes and analysis and will send feedback to PPI members on how they have shaped the analysis. Similarly, after Workshop three CMcG, AM, CC and KL will reflect on their own and PPI members’ interpretation of the data and provide feedback to PPI members on how they have shaped final stages of analysis.

All three of the workshops will be extensive and will require considerable investment in terms of time from PPI members. To enable PPI members to be engaged throughout each of the workshops academic researchers will ensure that breaks are scheduled.

## PPI Impacts

In the short term, we anticipate that impact relating to the PPI activities will include adapting data collection tools so that are inclusive of the public perspectives. Based on previous reports of PPI impact [26, 27] developing topic guides with the input from public contributors will enable them to be clear, concise and suitably worded.

Piloting the interviews will help the academic researchers further develop their own skills and confidence around interviewing techniques. For example, they may become more sensitised to how participants may feel when being interviewed. Working with PPI members when analysing the qualitative data could contribute to improving the overall rigour of the data analysis. The questions and discussions with the PPI members during this process could encourage continuous reflexivity, which in turn will enhance the trustworthiness of the findings [28]. Outlining the steps used within data analysis will promote transparency and openness on how analysis progressed and conclusions were drawn. Furthermore, and as described within Additional file 1, PPI will also contribute to the dissemination of findings both locally and nationally.

Critical reflections which include both positive and negative experiences of PPI are lacking. Once the PPI activities have concluded, the academic research team will meet to reflect on whether PPI activities met initial objectives. The academic researchers within this project will collect, reflect and share the findings from the Cube evaluation framework [23] to decipher if the workshops were positively received and where improvements can be made. The impact logs completed by the academic researchers after each workshop will be discussed within PPI study team meetings; this will encourage critical reflection of the PPI process and build team members’ capacity and skills in public involvement and research practices more generally.

We envisage that the long-term impacts of PPI activities within this project will be to increase personalised care for patients with multiple long-term conditions by ensuring that implementation barriers and facilitators identified by PPI contributors are taken into account. The PPI activities willlead to more focussed and relevant qualitative data collection and analysis that is driven by those who reflect the users of healthcare services. As a result, this will increase the real-world impact and success of healthcare research. Furthermore, considering the small number of studies on PPI in qualitative analysis, and the absence of methodologies for the co-production of data analysis, this PPI activity will provide a meaningful and transparent contribution to address the apparent knowledge gap. The wider academic community could learn from good PPI practice or identify aspects of improvement within their own PPI activities.

Implementation of research skills into practice has been identified as a key outcome of PPI training [29]. The PPI members involved in this study will have gained new knowledge and skills that they can take forward to future projects. PPI members could share their new skills and experiences of being involved in qualitative data collection and analysis with their peers spanning the knowledge exchange to a broader audience than those who attend the activities.

By creating a culture of shared learning and support, the authors hope that PPI members will gain new skills and confidence to challenge researchers’ ideas and assumptions. One of the impacts of the proposed PPI activities will be open and honest co-production where sincere thoughts and feelings can be shared and meaningful relationships are developed which may progress beyond the PP4M project. A summary of our proposed PPI activities are also reported using the GRIPP 2 Short Form in Additional file 2.

## Ethical considerations

Whilst UK ethics committee approval is not required for most PPI activities [30], the academic researchers will approach all PPI activities in an ethical manner. The self-selected demographic characteristics provided by PPI members will not be used as data in any form and will be kept confidential and stored securely on a Bristol University web-server. Audio-recordings of PPI activities will be used for note taking purposes only. Researchers will obtain verbal consent from all PPI members present at the start of the activity. The recordings will be destroyed as soon as notes have been written. When deciding whether to be involved in PPI activities, PPI members will be given forewarning of the intention to audio-record and will know the purpose, what will happen to the recording and content, and be able to object or withdraw.

As PPI members will be experiencing multiple long-term conditions, being involved in PPI activities may be triggering and academic researchers will make every effort to provide a safe environment where the person can be heard and supported as needed. Taking this into account, academic researchers will adhere to Mitchell et al.’s ethical PPI framework [31] which includes: prioritising PPI activities, agreeing language and working towards a shared understanding of tasks, gaining consent, maximising the benefits to public contributors, ensuring equity of access, providing researcher training, offering training to the public contributors and providing funding and recognition to PPI members. Whilst no formal collaboration agreement will be signed, within each PPI activity academic researchers will gain verbal consent from each public contributor.

Within each PPI activity, the ethical approach will be as follow:

1. To carefully prepare each session in advance, with specific information, tasks and/ or questions for the group in clear, accessible English.
2. To allow introductions and discussions about any experiences with multiple long-term conditions, if the public contributor should wish too.
3. Making it clear that the public contributor is under no obligation to take part in any element of PPI work for the project, and can leave the session at any time.
4. Ask for verbal consent from each public contributor at each meeting.
5. Provide written information about the study, nature of the PPI activity and contact details of the researchers.
6. Encourage public contributors to discuss their involvement with their peers.
7. Reassure public contributors that their contribution can remain anonymous.

Every effort will be made to provide a safe environment where the public contributor can be heard and supported as needed. Should any public contributor require support during the PPI activities, this can be provided by the academic researcher. If necessary, public contributors can be provided with information about whether to access further support. All of the PPI activities will include a burden of time on PPI members; they will be made aware that they can withdraw from any activity at any time.

After each PPI activity, academic researchers will meet to debrief and reflect on the activities; this may help to maintain rigour in the project [32]. Whilst the academic researchers currently have no plan to seek formal ethical approval from the appropriate research governance structures, they may decide to apply in the future when evaluating the involvement of this project or if they wish to use quotations from PPI members within a publication.

## Summary

This article has described proposed PPI activities relating to qualitative data collection and analysis embedded throughout an ongoing research project. The proposal has detailed the specific area of PPI activities (qualitative data collection and analysis), stated the aims of the PPI activities, provided a description of the proposed activities and documented the foreseen impact of PPI. By being open and transparent about PPI processes this article could help others planning similar activities.

## Supplementary Information

**Additional file 1**. Overall PPI plan.

**Additional file 2**. GRIPP 2 Short form.

## Data Availability

Not applicable.

## Abbreviations

ARC: Applied Research Collaborations
NIHR: National Institute for Health Research
PP4M: Personalised Primary care for Patients with Multiple long-term conditions
PPI: Patient and Public Involvement
RUG: Research User Group
UK: United Kingdom

## References

[1] INVOLVE. Briefing notes for researchers—PUBLIC involvement in NHS, health and social care research. (n.d.). Retrieved 16 August 2022, from https://www.nihr.ac.uk/documents/briefing-notes-for-researchers-public-involvement-in-nhs-health-and-social-care-research/27371.

[2] Heywang-Köbrunner SH, Hacker A, Sedlacek S (2011). Advantages and disadvantages of mammography screening. Breast Care.

[3] Brett JO, Staniszewska S, Mockford C, Herron-Marx S, Hughes J, Tysall C, Suleman R (2014). Mapping the impact of patient and public involvement on health and social care research: a systematic review. Health Expect.

[4] Ennis L, Wykes T (2013). Impact of patient involvement in mental health research: longitudinal study. Br J Psychiatry.

[5] Edelman N, Barron D (2016). Evaluation of public involvement in research: time for a major re-think?. J Health Serv Res Policy.

[6] Boote J, Baird W, Beecroft C (2010). Public involvement at the design stage of primary health research: a narrative review of case examples. Health Policy.

[7] A brief guide to public involvement in funding applications. Retrieved 13 August 2022, from https://www.nihr.ac.uk/documents/a-brief-guide-to-public-involvement-in-funding-applications/24162.

[8] Morgan H, Thomson G, Crossland N, Dykes F, Hoddinott P (2016). Combining PPI with qualitative research to engage ‘harder-to-reach’ populations: service user groups as co-applicants on a platform study for a trial. Res Involv Engag.

[9] Muller I, Santer M, Morrison L, Morton K, Roberts A, Rice C, Williams M, Yardley L (2019). Combining qualitative research with PPI: reflections on using the person-based approach for developing behavioural interventions. Res Involv Engag.

[10] Jennings H, Slade M, Bates P, Munday E, Toney R (2018). Best practice framework for patient and public involvement (PPI) in collaborative data analysis of qualitative mental health research: methodology development and refinement. BMC Psychiatry.

[11] Troya MI, Dikomitis L, Babatunde OO, Bartlam B, Chew-Graham CA (2019). Understanding self-harm in older adults: a qualitative study. EClinicalMedicine.

[12] Balazs CL, Morello-Frosch R (2013). The three Rs: how community-based participatory research strengthens the rigor, relevance, and reach of science. Environ Justice.

[13] Lovell, K. Enhancing the quality of service user involved care planning in Mental Health Services (EQUIP). The National Institute for Health and Care Excellence. Retrieved 23rd September 2022, from https://www.nice.org.uk/sharedlearning/enhancing-the-quality-ofservice-user-involved-care-planning-in-mental-health-services-equip.

[14] Cancer Research UK. Patient involvement toolkit for researchers. Retrieved from 23rd September 2022 from https://www.cancerresearchuk.org/funding-for-researchers/patient-involvement-toolkit-for-researchers. Accessed 19 Jan 2021.

[15] INVOLVE. Top tips. Retrieved on the 23rd November 2022 from: https://www.invo.org.uk/resource-centre/learning-and-development/top-tips.

[16] National Health Council. The National Health Council Rubric to Capture the Patient Voice: A Guide to Incorporating the Patient Voice into the Health Ecosystem. June 2019. Washington, DC. 2022. Retrieved 1st November 2022, from https://www.nationalhealthcouncil.org/Patient-Engagement-Rubric. Accessed 1 Nov 2021.

[17] Staniszewska S, Brett J, Simera I, Seers K, Mockford C, Goodlad S, Altman DG, Moher D, Barber R, Denegri S, Entwistle A (2017). GRIPP2 reporting checklists: tools to improve reporting of patient and public involvement in research. BMJ.

[18] Coldham T, IA Group. Guidance on co-producing a research project. (2018).

[19] National Development Team for Inclusion. The Core Principles of Co-Production. Retrieved 28th March 2023 from: https://www.ndti.org.uk/projects/the-core-principles-of-co-production.

[20] Oliver K, Kothari A, Mays N (2019). The dark side of coproduction: do the costs outweigh the benefits for health research?. Health Res Policy Syst.

[21] NIHR INVOLVE. UK Standards for Public Involvement- Better public involvement for better health and social care research [Online]: NIHR. Retrieved 1st August 2022 from: https://www.invo.org.uk/wp-content/uploads/2019/11/UK-standards-for-public-involvement-v6.pdf.

[22] People in Health West of England. Involvement Opportunities. Retrieved 19th April from: http://www.phwe.org.uk/involvement-opportunities/.

[23] Gibson A, Welsman J, Britten N (2017). Evaluating patient and public involvement in health research: from theoretical model to practical workshop. Health Expect.

[24] Hinton EC, Fenwick C, Hall M, Bell M, Hamilton-Shield JP, Gibson A. Evaluating the benefit of early patient and public involvement for product development and testing with small companies (2022). 10.1111/hex.13731 PMC10154839 36786161

[25] Kok M. Guidance document: evaluating public involvement in research. UWE Bristol e-prints repository 2018 [cited 2022 August]; Available from: http://www.phwe.org.uk/wp-content/uploads/Guidance-on-evaluating-Public-Involvement-in-research.pdf.

[26] Rouncefield-Swales A, Harris J, Carter B, Bray L, Bewley T, Martin R (2021). Children and young people’s contributions to public involvement and engagement activities in health-related research: a scoping review. PLoS ONE.

[27] Hyde C, Dunn KM, Higginbottom A, Chew-Graham CA (2017). Process and impact of patient involvement in a systematic review of shared decision making in primary care consultations. Health Expect.

[28] Ballinger C (2004). Writing up rigour: representing and evaluating good scholarship in qualitative research. Br J Occup Ther.

[29] Kristensen N, Nymann C, Konradsen H (2015). Implementing research results in clinical practice-the experiences of healthcare professionals. BMC Health Serv Res.

[30] Health Research Authority. Public Involvement. https://www.hra.nhs.uk/planning-and-improving-research/best-practice/public-involvement/.

[31] Mitchell SJ, Slowther A-M, Coad J, Akhtar S, Hyde E, Khan D, Dale J (2019). Ethics and patient and public involvement with children and young people. Arch Dis Child Educ Pract.

[32] Heckel M, Meesters S, Schildmann E, Ostgathe C (2020). Patient and public involvement (PPI) in palliative care research. Zeitschrift fur Evidenz, Fortbildung und Qualitat im Gesundheitswesen.

