# Supplementary material for: A proposal to embed patient and public involvement within qualitative data collection and analysis phases of a primary care based implementation study
Source: Res Involv Engagem. 2023 May 31;9:37. doi: 10.1186/s40900-023-00440-7 (PMC10233873; doi:10.1186/s40900-023-00440-7)
Supplement: Supplementary file 1 — Additional file 1. Overall PPI plan. [file 40900_2023_440_MOESM1_ESM.docx]

# PPI&E in PP4M

## Overall PPI plan

Our PPIE will build on active, diverse and established groups in each ARC area, that will continue into the project.

The Bristol PPI group provided significant input into project design, including template wording, content of the patient preparation letter and evaluation measures that reflect what is most important to patients. Their recommendations include that a review appointment should preferably be with a known clinician, and providing choice in the way the practice communicates and provides consultations. Simon Chilcott, who previously contributed to the 3D study, is a co-applicant and will be a core team member.

For the project we will arrange a public contributor group in each of the three collaborating sites. These sub-groups will consider, ways of encouraging patient engagement with the reviews, implementation strategies and adaptive strategies for particularly deprived or under-served areas. We may also work through community groups in areas of health inequity to recruit additional public contributors to advise on barriers to care and inform local implementation and engagement strategy. A PPIE Operational Group will lead overall PPIE strategy, chaired by the PPIE lead from ARC West and the lay co-applicant (SC). This Operational Group will collate feedback from the three local PPIE groups and will have a direct link into the Executive (Figure 1).

Figure 1


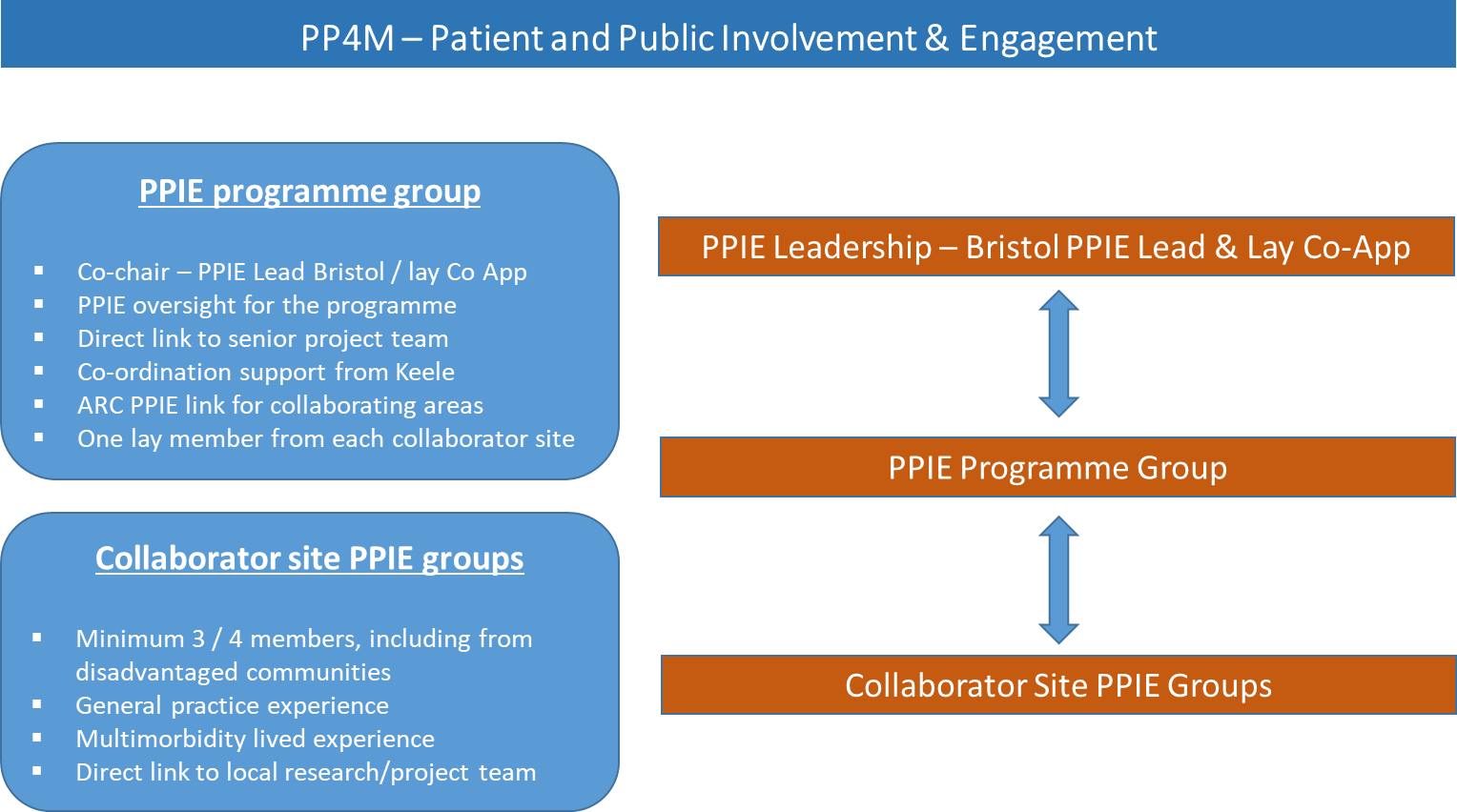


Local meetings will take place 3-4 times a year with a group selected in accordance with local strategies and protocols, but which includes people with lived experience of multimorbidity. These meetings should be timed around project milestones to maximise opportunity for PPIE input. Each area will evaluate their PPIE efforts, which will be used as a basis for an overall evaluation of the PPIE input. A standardised impact form will be used for this purpose.

Operational group meetings will take place twice a year and include the PPIE leads from each area plus a lay representative or champion from each area

PPI representation in core team meetings will be by Simon Chilcott, lay co-applicant, and the co-investigator PPI leads from each area.

Overall PPIE leadership will be from the lay co-app Simon Chilcott and the Bristol PPIE lead

Notes:

We recognise that involvement may be limited by the extent to which the template and accompanying IT can be changed and might be limited to advice on study documentation, community engagement to spread the idea of personalised reviews, and interpretation of responses. There has already been extensive involvement in the content of the template but not all of the suggestions will feature in the Arden’s template.

**PPIE Operational Structure:**

- Lay representation from the lay co-applicant will be provided to the PP4M Executive steering group. PPIE will be a standing agenda item on this meeting.
- A multisite PPIE operational group will provide the PPIE leadership to the project and will have membership from all project delivery sites (Wessex, Bristol and Keele)
- Individual PPIE groups will operate at each project delivery site (Wessex, Bristol and Keele) and will recruit lay members to reflect the study needs of each site.

**PPIE Governance:**

- Individual project delivery sites will convene their own PPIE to profile the needs of their local study population
- PPIE activity and impact will be captured by the Multisite PPIE operational group and reported into the PPIE leads meeting, who in turn report to the Steering Executive group.
- A record of PPIE involvement and activity will be maintained and shared at operational group level
- Each delivery site will follow their local NIHR ARC PPIE strategy

**PPIE Objectives:**

The core PPIE objectives for the project will include

- Recruitment of lay members at all project delivery sites to reflect the local population needs
- Co-production of an involvement plan for PPIE
- Lay review of study documentation
- Support data interpretation
- Co-production of a Primary Care implementation plan and support for its dissemination
- PPIE involvement in a shared webinar at the end of the project

## Table of PPI&E activities – core activities are in italics

| **Organisational tasks** | **Implementation activities** | **Research-related activities** |
| --- | --- | --- |
| *Recruit group of people with multimorbidity, attending to diversity and representation from areas of health inequality* | *Co-production of a primary care implementation plan* | *Review study documentation, including consent forms, ethics issues, invitation letters and format of questionnaires* |
| *Minimum meeting frequency every 4 months* | Consideration of adaptive strategies for particularly deprived or under-served areas | Recruitment – how to reach people, potential barriers, means of response |
| *Develop involvement plan with group* | Spread information among patient groups about implementation of the template promoting personalised care to create expectations and generate feedback | *Data interpretation - qualitative analysis and/or interpretation of findings* |
| *Maintain record of PPI involvement* | Ways of encouraging patient engagement with the reviews | PPI contribution to shared report |
| *Comply with shared plan for evaluation of PPI impact and process using agreed template* |  | PPI contribution to dissemination of findings local and nationally, including authorship of papers and speaking at meetings as appropriate and wished for |
| *PPI leads from each area meet twice a year* |  |  |
| *Invite PPI group from each area to shared webinar at end of project* |  |  |
